# #DryByChristmas: A patient and public involvement study on women's engagement with humorous pelvic floor muscle training digital nudges on social media

**DOI:** 10.1111/hex.14033

**Published:** 2024-03-31

**Authors:** Rosie C. Harper, Sally Sheppard, Elaine Miller, Carly Stewart, Carol J. Clark

**Affiliations:** ^1^ Faculty of Health and Social Sciences Centre for Midwifery and Women's Health Bournemouth University Poole UK; ^2^ University Hospitals Dorset NHS Foundation Trust Poole UK; ^3^ Chartered Society of Physiotherapy and Comedian London UK; ^4^ Faculty of Science and Technology Bournemouth University Poole UK

**Keywords:** patient and public involvement, pelvic floor training

## Abstract

**Introduction:**

Patient and public involvement (PPI) is essential for women's health research. Little is known about how women engage with humorous social media and behavioural health messaging targeting pelvic floor muscle training (PFMT). This PPI aimed to understand how women engage with a humorous social media campaign encouraging PFMT. The study findings will influence the co‐design of a digital intervention to support women's adherence to PFMT.

**Methods:**

The Guidance for Reporting Involvement of Patients and the Public Version 2 short form was used to report the study's findings. The study examined public engagement with a humorous social media campaign encouraging PFMT in women. A healthcare professional and comedian ran the campaign following the national guidelines for engagement in PFMT. Instagram analytics gave insight into the demographics of the public who engaged, how they engaged and the most popular content. The behaviour change techniques (BCTs) used in the digital nudges that generated the highest levels of engagement were analysed using the Capability Opportunity Motivation Behaviour Change Wheel.

**Results:**

The majority (96%) of the population showing the highest levels of engagement were women aged 35–44 years and were based in the United Kingdom (77%). The Instagram account saw an increase in engagement by 12% over the 3‐month campaign, with 22,032 users seeing digital nudges and 2645 engaging with the digital nudges. The preferred way of engaging was using Likes (9723). The common themes in the digital nudges that generated the highest levels of engagement were BCTs associated with the ‘social influences’ theoretical domain framework that targeted the core behaviour opportunity.

**Conclusion:**

The study findings suggest humour may improve women's engagement with online PFMT programmes; however, more rigorous research is required to better understand diverse women's experiences of humorous online PFMT nudges. Future studies may use PFMT mobile apps instead of social media to capture true user engagement and adherence to PFMT more accurately. The insights gained from the study will be taken forward to co‐design a digital behavioural intervention as part of a larger study.

**Public Contribution:**

Members of the public were involved in the co‐design of a digital health intervention that will be trialled as part of a larger research study. The public was involved using the social media platform Instagram. Public engagement with a humorous social media campaign to encourage women to engage with pelvic floor exercises was captured using Instagram analytics, for example, the timing of engagement.

## 1 INTRODUCTION

Patient and public involvement (PPI) is carried out ‘with’ or ‘by’ members of the public to improve research integrity, serve broader democratic principles and provide an opportunity for the public to influence how research is conducted.^1^ PPI involves the public in different ways to allow them to contribute to important decisions in the research process, such as providing feedback and suggestions on tools or interventions from a consumer perspective.^2^ Studies have shown PPI improves research quality, relevance and impact by increasing the likelihood of research translating into practice and improving population health^3^ by providing insights into the patient experience and highlighting barriers and facilitators to treatment adherence.^4^ A means of involving the public in research is through contribution to important decisions in the research process, such as the design of novel interventions.^3^

PPI is vital in women's health research because it engages a historically underserved population. As many as 84% of women in the United Kingdom felt they were not listened to by healthcare professionals when seeking treatment, according to the National Women's Health Strategy for England.^5^ Inviting women to actively engage with research processes and share their opinions on what is important to their health needs is essential when developing novel interventions to improve women's health outcomes.

Pelvic floor muscle dysfunction (PFMD) is a long‐term condition and public health issue that predominantly affects women following pregnancy and childbirth. Women are often reluctant to discuss or admit to having symptoms associated with PFMD, which may be considered taboo in some cultures. The most common symptom is urinary incontinence, and other symptoms include faecal incontinence, pelvic organ prolapse and pelvic pain. One in three women experience symptoms of PFMD at some point in their lifetime.^6^, ^7^

Pelvic floor muscle training (PFMT) is the gold standard of treatment for PFMD^8^ and requires women to practice the exercises daily over 3 months to benefit from the effects.^7^, ^9^ Self‐management of PFMT in the home reduces pain and disability^10^; however, women demonstrate poor adherence to PFMT,^11^ which means their symptoms persist, and they are required to manage using incontinence pads or frequent toilet visits. Adherence to a new exercise regime can be challenging, especially for women who have recently given birth and whose lives are taken up caring for a young family, and behavioural support is often necessary for women to engage effectively with PFMT.

Humour is a form of ‘persuasion’ according to the Capability Opportunity Motivation Behaviour Change Wheel (COM‐BCW).^12^ The COM‐BCW is a framework developed out of 19 behavioural theories. Humour has been used in public health campaigns to elicit favourable audience responses.^13^ However, the success of behavioural campaigns that use humour depends on how well‐understood the target population is.^12^, ^14^ How a population responds in a particular environment is key to designing more effective interventions.^15^ In the field of Nudge theory, there is a discussion about the involvement of persuasive cues and ‘environmental reconstruction’ to elicit ‘predictable’ changes in behaviour within a target population.^16^ These cues may be initiated through text, video or images and delivered through various media platforms.

The popular photo and video‐sharing social media site *Instagram* is a platform where humour can be shared internationally with immediate and broad reach. Compared to other popular social media platforms, Instagram has the highest levels of user engagement.^17^ Although women advocate for digital health technology,^18^ we know less about how they engage with public health campaigns on social media. Therefore, a better understanding of how women engage with a public behavioural campaign on social media that uses humour to support women in PFMT will contribute to designing more tailored digital interventions in the future.^12^

This PPI work aimed to better understand how women engage on Instagram in a humorous social media campaign. The goal was to encourage women to engage with PFMT and adhere to the training long enough to be effective. The #DryByChristmas campaign involved daily digital ‘nudges’ in the form of humorous social media posts that support women in their PFMT in line with the National Institute for Health and Care Excellence (NICE) guidelines.^7^

## 2 METHODS

### 2.1 Aim

This PPI work aimed to provide preliminary information on how women engaged with humorous digital PFMT nudges on Instagram as part of a social media campaign to promote PFMT. The Guidance for Reporting Involvement of Patients and the Public Version 2 short form checklist was used to enhance the quality, transparency and consistency of reporting the study.^4^, ^19^

### 2.2 PPI questions

1. What demographic of women engaged with the digital nudges on Instagram?
2. How did women engage with digital nudges that promoted PFMT?
3. Which digital nudges generated the highest levels of engagement?
4. What time of day are women most likely to respond to digital nudges on Instagram?
5. What are the behaviour change techniques employed in digital nudges?
6. What type of digital nudges do women prefer to engage with?
7. What behaviour change theories underpin the most popular digital nudges women engage with?

### 2.3 Public involvement

Instagram users were involved in the study. Individual Instagram users who viewed and/or engaged with the digital content as part of the #DryByChristmas campaign were included. The study captured how Instagram users engaged with the #DryByChristmas campaign.

### 2.4 The #DryByChristmas campaign

The social media campaign called #DryByChristmas was an online public health campaign that encouraged women to adhere to PFMT daily in line with NICE guidelines.^7^ Different images and videos were posted daily.

The #DryByChristmas campaign was run by E. M., a co‐author of the article. E. M. is a specialist women's health physiotherapist and comedian who uses humour to deliver evidence‐based key health messages around pelvic health issues. E. M. has years of experience working with women with PFMD and within the field of comedy. E. M. curated and posted all the photos and videos used in the campaign. E. M. chose the content of the posts based on her observations of women's responses in clinical practice and from her comedy performances. E. M. used her popular Instagram business account, @gusset_grippers, as a platform for the campaign.

### 2.5 Study duration

The social media campaign ran for 3 months, from October to December 2021. This time frame was based on current NICE guidelines^7^ and linked with the lead‐up to Christmas.

### 2.6 Capturing public engagement on instagram

Instagram's native analytics tool, ‘Insights’, was used to capture the behavioural insights of Instagram users in response to the social media campaign.

The engagement measures used were:

1. *Demographics*: The age and location of Instagram users.
2. *Engagement with the campaign*:
  (a) Total number of Instagram users who saw the images and videos posted as part of the campaign.
  (b) Total number of Instagram users who engaged with the posts through likes, comments, follows and visits to the @gusset_grippers Instagram profile.
3. *Timing*: When the photos and videos were most frequently seen and engaged with by followers of the @gusset_grippers Instagram account.
4. *Most popular posts*: Posts with the highest levels of user engagement (likes, comments, follows, profile visits).

### 2.7 Data extraction

The data was collected continuously during the campaign on Instagram's inbuilt behavioural analytics tool called ‘Insights’. Data were collected from Insights at the end of the campaign. The first author obtained permission and access to the @gusset_grippers business account to capture this data.

### 2.8 Data synthesis

The images and videos that generated the highest levels of engagement across each of the categories below were included in the analysis:

1. Number of comments left.
2. Number of followers gained.
3. Number of likes.
4. Number of profile visits.

Content that had the top three highest engagement levels under each category was included in the analysis. The image or video under each category with the highest levels of engagement was given a numerical value score (3, 2 or 1) and a corresponding Red Amber Green (RAG) rating.^20^ These scores were totalled to demonstrate which posts elicited the highest levels of engagement from the public across all categories. Total scores were calculated and presented using the same RAG system. Posts with a total RAG rating of one or more were analysed using the COM‐BCW.^12^

The COM‐BCW is a framework grounded in theory. It combines 19 behavioural theories and provides a standardised way of designing behavioural interventions based on the core components (capability, opportunity and/or motivation) researchers intend to target. The COM‐BCW does this by linking the theoretical models of behaviour to the theoretical domain framework.^12^ Using this well‐known method of designing interventions in reverse allows the identification of potential facilitatory behaviour change techniques (BCTs) in behavioural interventions. This method has been documented in other studies.^10^, ^21^

BCTs identified in the images and videos with a RAG rating of one or more were mapped to their core COM components using the COM‐BCW.^12^ The BCTs were identified by the first author. All recognised BCTs were included. All images and videos included one or more BCTs.

## 3 RESULTS

### 3.1 Demographics of users who engaged with digital nudges

Ninety‐six percent of the audience who engaged with the posts were women. Women aged 35–44 had the greatest engagement level (36%). Seventy‐seven percent of users who engaged were from the United Kingdom. Demographic data are summarised in Table 1.

**Table 1:** Demographics of the Instagram users who engaged with the #DryByChristmas campaign.

Demographic characteristics	Demographic data
Gender	96% female
Age	36% of the population were aged between 35 and 44 years
	25% of the population was aged between 45 and 54 years
	21% of the population were aged between 25 and 34 years
	11% of the population were aged between 55 and 64 years
Location	77% were from the United Kingdom
	5% the United States
	5% Australia
	4% Ireland

### 3.2 Women's preferred form of engagement with digital nudges

A total of 22,032 users saw the digital nudges posted by the #DryByChristmas campaign. Of 22,032, 6145 were followers of the @gusset_grippers Instagram account.

Of 22,032 users who saw the digital nudges, 2645 engaged. Of 2645, the majority followed the @gusset_grippers account (2236).

Most users who engaged with the campaign did so using Likes (9,723), followed by visiting the @gusset_grippers Instagram profile (4,862) and commenting on the posts (892). Far fewer engaged by following @gusset_grippers profile.

### 3.3 Digital nudges and levels of engagement

#### 3.3.1 Likes

Video 3 had the highest number of likes (4638). Video 8 had the second‐highest number of likes (3370), and Video 9 had the third highest (3198).

#### 3.3.2 Comments

Image 1 had the highest number of comments (85). Image 7 generated 49 comments. Image 2 had the third‐highest number of comments at 36.

#### 3.3.3 Number of followers gained

Video 5 generated the highest number of new followers (16), followed by Image 4 (12) and Image 6 (6).

#### 3.3.4 Profile visits

Image 1 generated the highest levels of profile visits (86), followed by Video 3 (55) and Image 7 (54).

The digital nudges with the highest levels of engagement were given a score of 3, the second highest of 2 and the third a score of 1. A table of the most popular images and videos is presented in Table 2. The total levels of engagement can be seen in Tables 3 and 4.

**Table 2:** The images and videos that generated the highest levels of engagement in the #DryByChristmas campaign.

Image 1	Image 2	Video 3
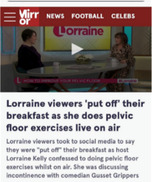	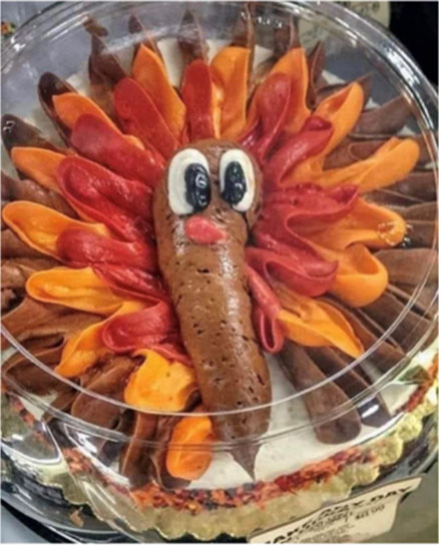	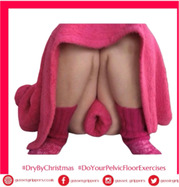
Image 4	Video 5	Image 6
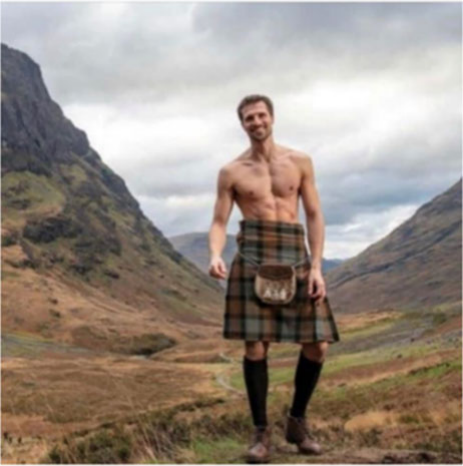	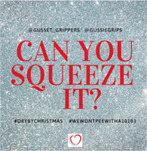	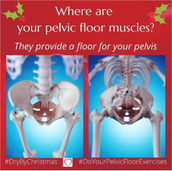
Image 7	Video 8	Video 9
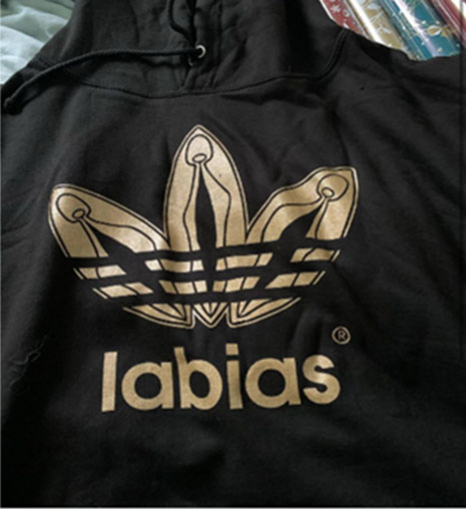	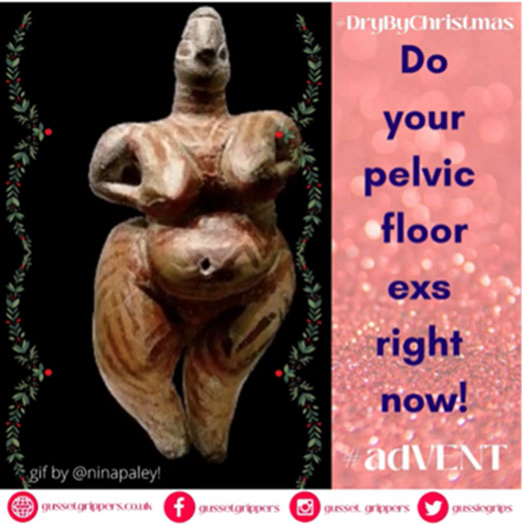	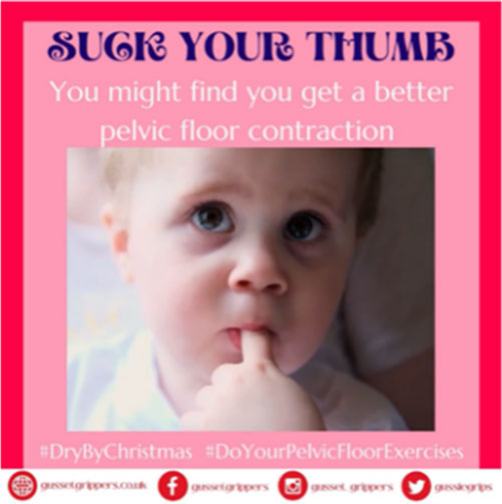

**Table 3:** Levels of engagement on the most popular digital reminders used in the #DryByChristmas campaign.

Digital reminder	Number of comments	Number of followers gained	Number of likes	Number of profile visits	Total score
Image 1	3			3	6
Image 2	1				1
Video 3			3	2	5
Image 4		2			2
Video 5		3			3
Image 6		1			1
Image 7	2			1	3
Video 8			2		2
Video 9			1		1

*Note*: The post with the highest levels of engagement under each category will be given a score of 3, followed by a score of 2 for the second highest and 1 for the third highest. These scores were totalled to demonstrate which posts elicited the highest levels of engagement from women across all categories. A corresponding Red Amber Green colour rating was given where green shows the highest levels of engagement.

**Table 4:** Bar chart representing the levels of engagement on each digital reminder according to Red Amber Green rating.

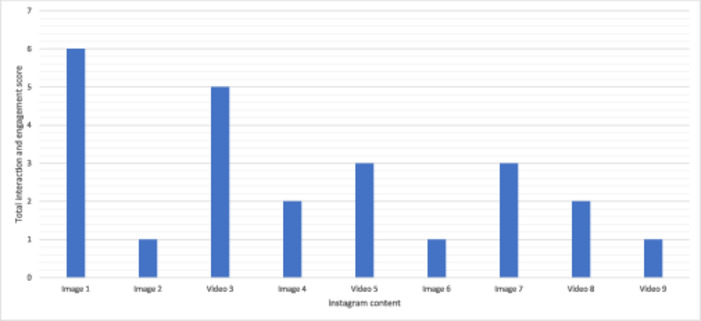

#### 3.3.5 Timing of digital nudges

Women were most active at 6 o'clock in the evening. Women remained consistently active at 6 o'clock during weekdays and weekends.

#### 3.3.6 Most popular posts

Image 1 had the highest levels of engagement across all categories, with a score of 6, as seen in Table 3. Video 3 had the second highest levels of engagement at 5. Video 5 and Image 7 scored 3 and generated the third‐highest levels of engagement. The content of these images and videos went on to be analysed.

#### 3.3.7 Behaviour change techniques used in digital reminders with the highest engagement

The BCTs identified in the digital nudges and the associated core behaviour can be seen in Table 5. BCTs identified included framing/reframing, social support, information about others’ approval, credible sources, prompt/cue, self‐incentive, goal‐setting outcome, instruction on how to perform the behaviour and valued self‐behaviour. The most targeted core behaviour that elicited the greatest engagement was ‘opportunity’, followed by ‘motivation’ and ‘capability’.

**Table 5:** Behaviour change techniques identified and their corresponding target behaviour in digital reminders with the highest levels of engagement.

Behaviour change technique	Behaviour change taxonomy grouping	Associated theoretical domains framework	COM‐B component identified in behaviour analysis	COM core behaviour targeted
Framing/reframing	Identity	Social influences and optimism	Social opportunity and reflective motivation	Opportunity and motivation
Social support	Social support	Social influences	social opportunity	Opportunity
Information about others' approval	Comparison of behaviour	Social influences	Social opportunity	Opportunity
Credible source	Comparison of outcomes	Beliefs and consequences	Reflexive motivation	Motivation
Prompt/cue	Association	Environmental context and resources	Physical opportunity	Opportunity
Self‐incentive	Reward and threat	Reinforcement	Automatic motivation	Motivation
Goal‐setting outcome	Goals and planning	Goals	Reflexive motivation	Motivation
Instruction on how to perform behaviour	Shaping knowledge	Skills and knowledge	Psychological and physical capability	Capability
Valued self‐identity	Identity	Social influences	Social opportunity	Opportunity

Abbreviation: COM‐B, capability opportunity and motivation behaviour.

#### 3.3.8 Discussion and critical reflection

While there is a substantial body of work on the effectiveness of PFMT, adherence remains a challenge. This PPI work is the first to consider the role of humour and behavioural change mechanisms via a comedic social media campaign to enhance adherence to PFMT. The intersection of humour, pelvic floor health and social media is a novel space offering valuable insights into alternative methods for public engagement and adherence to PFMT. Research has shown that incorporating humour into education and training can improve engagement and motivation, reduce stress and improve mood.^22^, ^23^ Engaging the public using comedy is an alternative to conventional engagement methods and may help alleviate embarrassment or discomfort associated with pelvic floor issues and performing the exercises. Our analysis indicates that this approach may have created a more relaxed and open environment, encouraging individuals to participate without feeling self‐conscious and normalising the conversation about pelvic floor health.

Women of childbearing age demonstrated the greatest levels of engagement with the #DryByChristmas campaign. One reason could be that PFMT is a higher priority for this population. Pregnancy and childbirth increase the risk of women developing symptoms of PFMD.^7^, ^24^ Women of childbearing age may have engaged with the campaign because they have symptoms or because they have become more aware of the pelvic floor through contact with healthcare professionals during pregnancy. Perinatal women are progressively seeking health‐related information from online resources, including social media.^25^, ^26^ The rising popularity of digital health is attributed to affordability and accessibility, irrespective of location.^27^ A systematic review and meta‐analysis examining the effectiveness of social media and mobile health apps in pregnancy found that out of 15 international randomised controlled trials, there was a moderate to strong effect on physical health outcomes.^28^ Therefore, targeted digital PFMT nudges may have a greater impact on PFMT engagement in studies involving perinatal women.

The digital PFMT nudges that generated the highest levels of engagement used behaviour change techniques associated with the ‘social influences’ theoretical domains framework. This suggests digital PFMT nudges that target interpersonal processes are more likely to influence how women feel and think about their pelvic floor health. Other studies have identified social influence as a key facilitator of behaviour change around diet adoption.^29^ In contrast, a scoping review found that social support showed some or no association with adherence. However, in this study, only one study that looked at women with urinary incontinence was found, which was of low quality.^10^

One reason social influence may have influenced women's behaviour towards PFMT is because it contrasts the isolation so many women experience with PFMD.^30^ The symptoms of PFMD have significant negative social, emotional, economic, and environmental impact.^30^ Socially, symptoms of PFMD prevent women from leaving the house and being intimate with their partner and create a barrier to physical activity that leads to distress and decreased quality of life.^30^ The use of social media publicly demonstrates through engagement how many women can relate to the information in the digital nudges. Public understanding of the shared experience of PFMD and self‐management options may inspire change and lead to a change in behaviour. These insights could be used to develop digital interventions to support women with PFMD.

The study findings suggested that using humour to support women's PFMT promoted engagement. Other populations have demonstrated positive health behaviours regarding using humour in social support.^31^ A possible reason for this is that humour challenges individuals’ attitudes towards taboo health topics on a cognitive level. Arguably, humour ‘bursts’ the taboo by creating a strong counter‐narrative against the silence the taboo creates.^32^ This powerful counternarrative that humour provides may empower people who previously felt alone and isolated by their PFMD symptoms.

On the other hand, humour in health messaging could be contentious. Although humour may potentially contribute positively to adherence, it is important to note that humour should be approached with cultural sensitivity and awareness. How humour is received is subjective and audience‐dependent.^33^ Most women who engaged with the campaign were from the United Kingdom (77%); however, humour and the use of a Christian holiday in the campaign may not translate to other cultures internationally and may cause exclusion. Understanding your audience increases the chance of delivering appropriately framed health information that will ultimately lead to greater adherence,^13^ which is critical when designing behavioural interventions.^34^ As a result, diverse women's voices will be included in a larger research project to better understand women's experience of digital PFMT nudges. Future PPI work could explore cross‐cultural humour conventions in pelvic floor health and inclusivity.

While the study demonstrated high levels of engagement with the #DryByChristmas campaign, there were several technical challenges when uploading content daily, and there was no opportunity to explore whether women's engagement with the #DryByChristmas campaign meant women completed their PFMT. This highlights the complexity of measuring behaviour change around exercise adherence remotely. The findings of this study, however, may influence the design of future digital health messaging to support women with PFMT remotely and change how patient exercise adherence in clinical settings is monitored and supported by clinicians. Behaviour change involves many interrelated factors, of which nudging is only one small part.^35^ Similarly, adherence depends on individual self‐efficacy and the number of prescribed exercises completed.^21^ More rigorous means of measuring women's behaviour and adherence to PFMT are required to better understand how PFMT digital nudges influence adherence. PFMT mobile apps on the NHS library provide an alternative means of distributing targeted health messaging at a population level^12^ while rigorously recording adherence using in‐app algorithms.

## 4 CONCLUSION

The study findings suggest humour may improve women's engagement with online PFMT programmes. However, more rigorous research is required to better understand diverse women's experiences of humorous online PFMT nudges. Future studies may choose to use PFMT mobile apps to capture true user engagement and adherence more accurately with PFMT. Insights gained from the study will be taken forward to co‐design a digital behavioural intervention as part of a larger study.

## AUTHOR CONTRIBUTIONS

**Rosie C. Harper**: Writing—original draft; writing—review and editing; investigation; formal analysis; data curation; methodology; conceptualization. **Sally Sheppard**: Writing—review and editing; supervision. **Elaine Miller**: Writing—review and editing; conceptualization. **Carly Stewart**: Writing—review and editing; supervision. **Carol J. Clark**: funding acquisition; writing—review and editing; supervision.

## CONFLICT OF INTEREST STATEMENT

The authors declare no conflict of interest.

## ETHICS STATEMENT

Ethical approval is not required for public and patient involvement and engagement activity.

## Data Availability

The data that support the findings of this study are available from the corresponding author upon reasonable request.
